# Poor Separation of Clinical Symptom Profiles by DSM-5 Disorder Criteria

**DOI:** 10.3389/fpsyt.2021.775762

**Published:** 2021-11-29

**Authors:** Jennifer Jane Newson, Vladyslav Pastukh, Tara C. Thiagarajan

**Affiliations:** Sapien Labs, Arlington, VA, United States

**Keywords:** diagnosis, symptom profiles, DSM-5, comorbidity, heterogeneity, depression, ADHD, autism spectrum disorder (ASD)

## Abstract

Assessment of mental illness typically relies on a disorder classification system that is considered to be at odds with the vast disorder comorbidity and symptom heterogeneity that exists within and across patients. Patients with the same disorder diagnosis exhibit diverse symptom profiles and comorbidities creating numerous clinical and research challenges. Here we provide a quantitative analysis of the symptom heterogeneity and disorder comorbidity across a sample of 107,349 adult individuals (aged 18–85 years) from 8 English-speaking countries. Data were acquired using the Mental Health Quotient, an anonymous, online, self-report tool that comprehensively evaluates symptom profiles across 10 common mental health disorders. Dissimilarity of symptom profiles within and between disorders was then computed. We found a continuum of symptom prevalence rather than a clear separation of normal and disordered. While 58.7% of those with 5 or more clinically significant symptoms did not map to the diagnostic criteria of any of the 10 DSM-5 disorders studied, those with symptom profiles that mapped to at least one disorder had, on average, 20 clinically significant symptoms. Within this group, the heterogeneity of symptom profiles was almost as high within a disorder label as between 2 disorder labels and not separable from randomly selected groups of individuals with at least one of any of the 10 disorders. Overall, these results quantify the scale of misalignment between clinical symptom profiles and DSM-5 disorder labels and demonstrate that DSM-5 disorder criteria do not separate individuals from random when the complete mental health symptom profile of an individual is considered. Greater emphasis on empirical, disorder agnostic approaches to symptom profiling would help overcome existing challenges with heterogeneity and comorbidity, aiding clinical and research outcomes.

## Introduction

The mental health of our society is a problem of growing concern. In 2017, 792 million people lived with a mental health disorder globally (1), while depression is the leading cause of disability as measured by Years Lived with Disability (YLDs) and a major contributor to the global burden of disease (2, 3). As people grapple with the consequences of the Covid-19 pandemic, the number reporting challenges with their mental health has further increased (4–7), emphasizing the importance of improving our understanding of mental illness to enable better outcomes.

In the absence of an understanding of underlying etiologies and biology of mental health challenges, the classification systems of DSM-5 (8) and ICD-11 (9) evolved to define mental health disorders by symptom criteria whereby specific groupings of symptoms are each assigned “disorder” labels. This approach presupposes that (i) the specific groupings of symptoms are good at separating individuals based on their symptom profile, such that individuals with a particular diagnosis have similar symptom presentations and (ii) that these symptom-based diagnostic groups each share a common underlying etiology. However, a large literature now highlights major misalignments between these disorder classifications and the symptomatic experience of patients. Firstly, the criteria-based approach to diagnosis, where one must have a subset of symptoms out of a larger group, means there are many ways to be diagnosed with the same disorder. For example, by some estimates there are 636,120 different possible symptom combinations that can lead to a diagnosis of PTSD (10) and 227 different possible ways to be diagnosed with depression (11), indicating considerable heterogeneity in symptom profiles within disorders. The observed heterogeneity in symptom profiles within disorders (12–19) has also led to various new definitions of disorder subtypes (20, 21). Secondly, there are many possible ways that patients can be comorbid across DSM-5 disorders (22), with studies showing that individuals commonly meet the criteria for multiple disorders (23–31) and that evolution of disorders across a lifetime is a pervasive phenomenon (28, 32–34). This misalignment is further exacerbated by an array of mental health assessment tools that are heterogeneous and overlapping, creating a system of diagnosis and evaluation that is poorly standardized and introducing further ambiguity (35, 36).

As a result, there has been considerable discussion over the validity of the DSM-5 classification approach (37–46). In addition, numerous studies have reported on the consequences of this misalignment between disorder classifications and patient symptom profiles. First, by focusing on a specific subset of symptoms for the definition of a disorder, this classification system precludes an understanding of the true range and diversity of symptom profiles present across clinical populations as the objective is typically to narrow down to a single disorder diagnosis, rather than embrace individual differences (47) or symptom complexity (48–50). However, the nuances of an individual's symptom profile hold important information that can guide clinical decision-making (51–58). Second, it results in inaccurate, not otherwise specified (NOS) or mixed diagnoses (59–63) where those with symptom profiles that are a poor fit for the clinical criteria, may have to embark on a long struggle to find effective treatment (64). Third, from a research perspective, studies aiming to develop new therapies and medications for mental health disorders typically select participants based on a diagnosis, whereas this group may be substantially heterogeneous in terms of their symptom profiles, and therefore outcomes (19, 21). To try to overcome some of these challenges, alternative transdiagnostic frameworks such as the Research Domain Criteria (RDoC) from the National Institute of Mental Health (NIMH) (65, 66) and the Hierarchical Taxonomy of Psychopathology (HiTOP) (67–69) have been proposed. These offer alternative ways to evaluate symptom profiles, either in terms of specific transdiagnostic constructs and subconstructs (RDoC), or dimensions at multiple levels of hierarchy (HiTOP), where the peak of the hierarchy can be denoted as a single overall factor of psychopathology, or p-factor (70, 71).

However, despite these various criticisms, the disorder classification system laid out by the DSM-5 is ingrained in psychiatric decision-making, social policy and popular discourse, and continues to dominate the field of mental health. One reason for the continued debate, is that there has not been a clear empirical assessment of how well the disorder classification system separates individuals into groups based on symptom criteria. For example, although there may be many combinations of symptoms that deliver a diagnosis of any individual disorder (10, 11), the overall symptom profiles of individuals with one diagnosis may nonetheless be substantially different relative to individuals in another disorder group. If so, the classification system would then serve to broadly discriminate between groups, which has a first order utility for differential determination of treatment pathways.

Here we assessed quantitatively the degree to which the symptom profiles of individuals associated with one disorder classification, as determined by the DSM-5 symptom criteria, were distinct from those of another, and how much they differed from a random selection of individuals with any disorder. To do so, we used a self-report mental well-being assessment tool, the Mental Health Quotient (MHQ) (72) that comprehensively covers mental health symptoms pertaining to 10 common DSM-5 disorders in a manner that is easy to administer, and on completion provides individuals with an aggregate score (quotient) along with a detailed report with feedback and recommendations for help seeking and self-care.

The MHQ was developed through a systematic analysis of the question content from 126 commonly used mental health questionnaires and interviews which typically conform to criteria laid down in the DSM (35). The 126 assessments included commonly used diagnostic scales and assessments of depression, anxiety, bipolar disorder, attention-deficit/hyperactivity disorder (ADHD), posttraumatic stress disorder (PTSD), obsessive compulsive disorder (OCD), addiction, schizophrenia, eating disorder, and autism spectrum disorder (ASD), as well as cross-disorder tools. Forty-three identified symptom categories were then combined with additional elements from RDoC to develop a questionnaire that comprehensively assessed a complete profile of 47 mental elements using a 9-point life impact rating scale [for details on the question format see (72) and Supplementary Figures 1A,B]. In this study, we used data from 107,349 respondents across 8 English-speaking countries (73) who completed the MHQ between April 2020 and June 2021. The 47 MHQ elements were mapped to the diagnostic criteria, as outlined in DSM-5, for the 10 mental health disorders on which the MHQ was based. We then evaluated the heterogeneity of symptom profiles of those within DSM-5 disorder groups relative to between disorder groups and random groupings of individuals who met the criteria for any one of the 10 disorders.

We show here that, contrary to expectation, the heterogeneity of the symptom profiles across individuals selected randomly across any disorder group, was no different than across individuals selected from within any specific disorder group. Furthermore, symptom profiles were as heterogeneous within a disorder group as they were between disorder groups. This challenges the fundamental premise on which the DSM-5 has been developed and has significant implications from both a clinical and research perspective.

## Materials and Methods

### Data Acquisition

The data sample was taken from the Mental Health Million open-access database which acquires data by offering the MHQ online and free of charge in multiple languages (72, 73). Respondents took this 15–20-min assessment anonymously online for the purpose of receiving a mental well-being score (the mental health quotient) and personalized report on completion, based on their responses (see below and Supplementary Figure 1C). The MHQ was publicized primarily through social media and Google Ads targeting a broad cross section of adults aged 18 and above, where self-selecting respondents may have had a specific mental health interest or concern. Outreach directed participants to the MHQ website (https://sapienlabs.org/mhq/) to complete the assessment. No financial compensation was provided. The Mental Health Million Project is a public interest project that tracks the evolving mental well-being of the global population, and its social determinants, and is governed by an academic advisory committee. The project has ethics approval from the Health Media Lab Institutional Review Board (HML IRB), an independent IRB that provides assurance for the protection of human subjects in international social & behavioral research (OHRP Institutional Review Board #00001211, Federal Wide Assurance #00001102, IORG #0000850).

For this study, we utilized responses to the English version of the MHQ between April 2020 and June 2021 which included 107,349 respondents predominantly from the United States, Canada, United Kingdom, Australia, New Zealand, South Africa, Singapore and India, with the majority of respondents living in India (26.35%) United States (25.68%), and smaller proportions of respondents living in Singapore (1.53%) and New Zealand (3.60%). Across all countries, 40.35% of the sample were male; 57.94% female; 0.95% reported as non-binary/third gender and 0.77% preferred not to say. The sample covered all age brackets but had larger samples in the younger (18–24; 22.50%) and older (55–64; 19.01%) age groups relative to their proportion in the population. These differences likely reflect the self-selected nature of the sample (see Limitations section in the Discussion). The demographic breakdown (including age, gender, country, employment status, and education) of the sample is shown in Table 1 and Supplementary Table 1.

**Table 1:** Demographic breakdown of the sample by age, gender, and country.

	***N***	**%**
**Age group**		
18–24 years	24,149	22.50%
25–34 years	12,740	11.87%
35–44 years	9,927	9.25%
45–54 years	15,314	14.27%
55–64 years	20,402	19.01%
65–74 years	17,741	16.53%
75–84 years	6,337	5.90%
85+ years	739	0.69%
**Gender**		
Males	43,313	40.35%
Females	62,195	57.94%
Non-binary/third gender	1,016	0.95%
Prefer not to say	825	0.77%
**Country**		
United States	27,571	25.68%
United Kingdom	16,115	15.01%
Australia	7,413	6.91%
Canada	8,012	7.46%
India	28,285	26.35%
New Zealand	3,866	3.60%
Singapore	1,639	1.53%
South Africa	7,804	7.27%
Other countries	6,644	6.19%

### MHQ Assessment

The MHQ is a transdiagnostic assessment that comprehensively covers all possible symptoms across 10 major mental health disorders as well as elements derived from RDoC constructs and subconstructs. The development and structure of the MHQ are described in previously published papers (35, 72). Briefly, the list of MHQ elements or items was determined based on a comprehensive coding of mental health symptoms assessed in questions across 126 different mental health questionnaires and interviews spanning 10 major mental health disorders as well as cross-disorder assessments. These included questionnaires for depression [e.g., Patient Health Questionnaire, PHQ-9 (74)], anxiety [e.g., Generalized Anxiety Disorder Assessment, GAD-7 (75)], bipolar disorder [e.g., Mood Disorder Questionnaire, MDQ (76)], ADHD [e.g., Conners Adult ADHD Rating Scale, CAARS (77)], PTSD [e.g., Clinician-Administered PTSD Scale for DSM-5, CAPS-5 (78, 79)], OCD [e.g., Yale Brown Obsessive Compulsive Symptom Scale & Checklist™, Y-BOCS (80, 81)], addiction [e.g., Addiction Severity Index, ASI-5 (82)], schizophrenia [e.g., Brief Psychiatric Rating Scale, BPRS (83, 84)], eating disorder [e.g., Eating Disorder Inventory, EDI-3 (85)], and ASD [e.g., Autism Diagnostic Interview Revised, ADI-R (86, 87)], as well as cross-disorder tools [e.g., Structured Clinical Interview for DSM-5, SCID-5-CV (88); see (72) for more details and a full list of the 126 assessment tools]. These disorders were selected based on their inclusion in the DSM-5 clinical interview (SCID-CV) (88). In addition, ASD and eating disorder were included due to both their prevalence and their broad public and scientific interest. A total of 10,154 questions were coded and consolidated into a set of 43 symptom categories. The resultant items were then reviewed in the context of other transdiagnostic frameworks including RDoC constructs and subconstructs put forward by the NIMH (65), and a few additions (e.g., selective attention, coordination) were made to ensure that the list of items reflected components within this non-DSM framework. The resulting categories were then reorganized into a set of 47 elements that describe mental health and mental well-being (**Figure 3** and Supplementary Table 2).

Within the MHQ, each of these 47 elements were rated by respondents using a 9-point life impact rating scale reflecting the impact of a particular mental aspect on one's ability to function (Supplementary Figures 1A,B). This scale was designed depending on whether the item exists on a spectrum from positive to negative (spectrum items such as memory) or as varying degrees of problem severity (problem items such as suicidal thoughts). For spectrum items, 1 on the 9-point scale referred to “Is a real challenge and impacts my ability to function,” 9 referred to “It is a real asset to my life and my performance,” and 5 referred to “Sometimes I wish it was better, but it's ok.” For problem items, 1 on the 9-point scale referred to “Never causes me any problems,” 9 referred to “Has a constant and severe impact on my ability to function,” and 5 referred to “Sometimes causes me difficulties or distress but I can manage.” Respondents made rating responses based on their current perception of themselves rather than a specific time frame. However, the rating on the 9-point life impact scale has been shown to have a good correlation against more common metrics of symptom frequency and severity, and ratings across elements are highly reliable from sample to sample (89).

Data across 30 descriptors relating to the demographic, life experience, lifestyle and situational profile of the individual were also collected. Demographic descriptors relating to the age, gender, geographical location, employment status, and education of the individual were obtained prior to the collection of mental well-being ratings, while life experience (e.g., life traumas, Covid-19 impacts, medical conditions, life satisfaction, substance use), lifestyle (sleep, exercise and socializing), and help seeking (including reasons for/ not) were obtained after the collection of mental well-being ratings [see (73) for a complete list].

On completion, respondents received their MHQ (a composite mental well-being score that places them along a spectrum from distressed to thriving), along with a personal report that provided recommendations for help-seeking and self-care. Provision of a personal report aimed to ensure greater interest of the respondent to answer questions thoughtfully and accurately. An extract of an example MHQ report detailing the MHQ score and sub-scores is presented in Supplementary Figure 1C; see (72, 73) for further information on how these scores were calculated. The MHQ score has substantial criterion validity, relating systematically to both productivity and clinical burden (89).

### Exclusion Criteria

The following exclusion criteria were applied to the data. First, those respondents who took under 7 min (an indication that the questions were not actually read) or over 1 h to complete the assessment (suggesting that the individual was not focused on the assessment), were excluded. Secondly, individuals who found the assessment hard to understand (i.e., responded *no* to the question, “Did you find this assessment easy to understand?”) were excluded. Third, respondents who made unusual or unrealistic responses (e.g., those who stated they had not eaten for 48+ hours or who stated they had slept for 30+ hours) were excluded. These responses might be poorly considered by the respondent or reflect circumstances where thinking was impaired and therefore invalid. Altogether, this resulted in the exclusion of 6.9% of respondents, with 107,349 respondents available for the final analysis.

### Symptom Criteria

For each of the 47 MHQ items, responses were determined to be clinically significant symptoms if they met a particular threshold of impact on the individual's ability to function (hereafter referred to as “clinical symptom(s)” or “symptom(s)”). For problem items which represented a unidimensional scale of symptom severity from 1 to 9, the threshold selection for a clinical symptom was ≥8. For spectrum items where elements of mental function could be either a negative symptom or a positive asset (e.g., memory) and the 1–9 scale ranged from negative symptom (1–4) to positive asset (6–9), the threshold selection for a clinical symptom was a rating of ≤1. Testing of the 9-point scale for one problem element (*Feelings of Sadness, Distress or Hopelessness*) demonstrated that a selection of 8 corresponded to an average symptom frequency of 5 days per week (89). This threshold thus corresponds to the DSM-5 criteria of experiencing the symptom “nearly every day” for depression. However, we note that the correspondence between frequency and life impact rating may differ from item to item (see Limitations section of the Discussion).

### Computing Dissimilarity of Symptom Profiles

**Figure 1:**
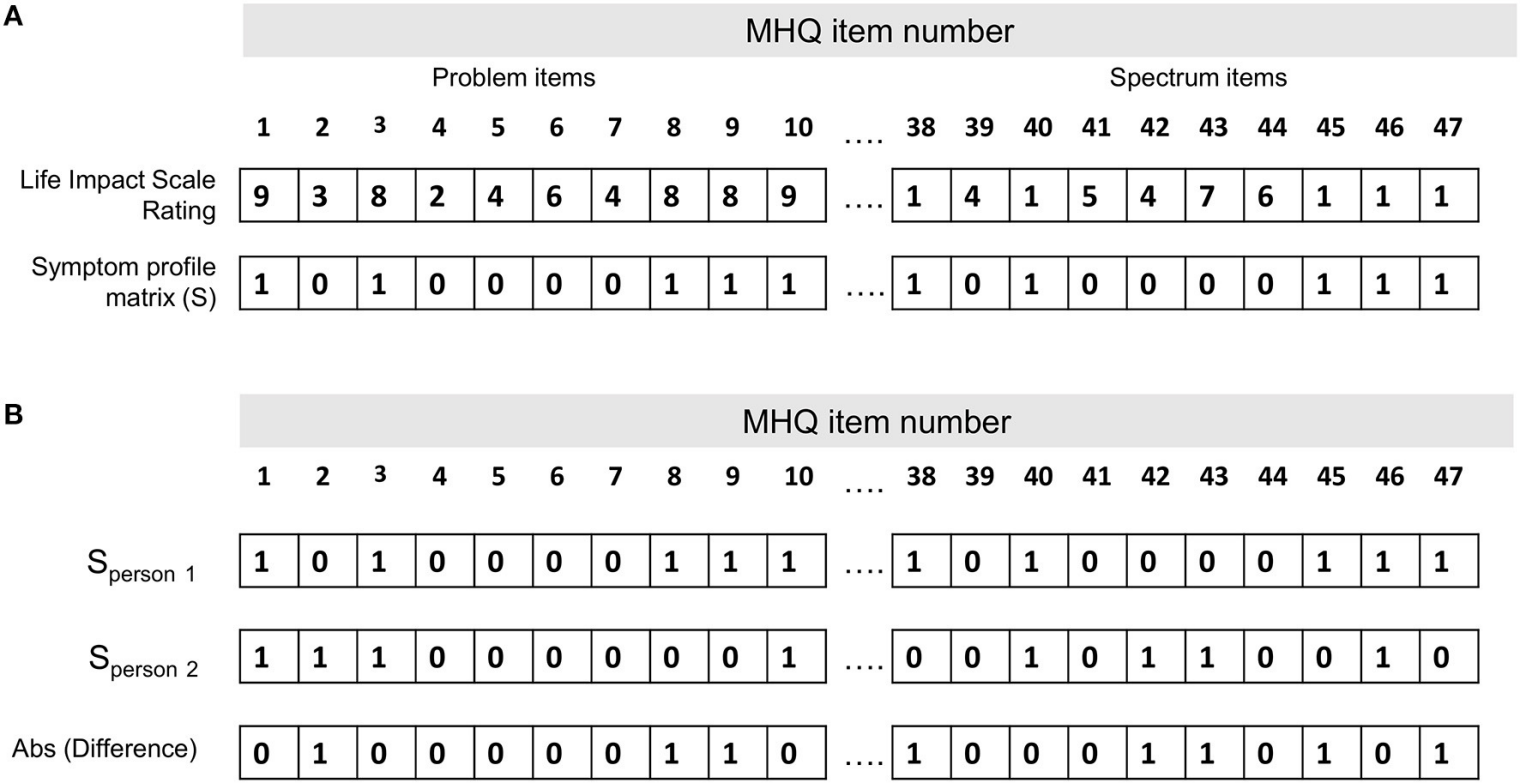
Calculation of the symptom dissimilarity between two people. **(A)** Symptom profile for one person (person 1 in **B**). Each of the 47 MHQ elements (representing a comprehensive set of possible mental symptoms) are coded as 1 if it is a symptom (i.e., the rating selected is above the threshold where it is considered a symptom, generally in this paper ≥8 for problem items and ≤1 for spectrum items), and 0 if it is not a symptom (i.e., does not meet the threshold). **(B)** Symptom profile for two people. Symptom dissimilarity is calculated as the sum of the absolute difference between the symptom matrix for each person/total number of symptoms.

Symptom dissimilarity between each pair of individuals was calculated as the percentage of symptoms that differed (i.e., where one person had the symptom and the other did not) out of the total number of possible symptoms (i.e., 47 MHQ items representing 47 possible symptoms; Supplementary Table 2). Figure 1A shows the symptom profile determined for one person whereby each of the 47 MHQ elements (representing a comprehensive set of possible symptoms) are coded as 1 if it was a symptom (i.e., the rating selected was above the threshold where it was considered a symptom, generally in this paper ≥8 for problem items and ≤1 for spectrum items), and 0 if it was not a symptom (i.e., did not meet the threshold). Symptom dissimilarity between two people was calculated as the sum of the absolute difference between the symptom matrix for each person as a fraction of the total number of symptoms (Figure 1B). Restated:


$$\begin{array}{l} \text{Symptom}\;\text{dissimilarity}=\left(\overset{N}{\underset{MHQ=1}{\sum }}Abs\left(Sp1-Sp2\right)\right)/N \end{array}$$

where Sp1 is the symptom profile of person 1, Sp2 is the symptom profile of person 2 and N is the total number of possible symptoms (here 47).

For example, out of 47 possible symptoms, if each person had 20 symptoms which did not overlap at all they would have a dissimilarity of 40/47, or 85%. If they each had just 10 symptoms which did not overlap, they would have a dissimilarity of 20/47, or 43%. Conversely, if all 10 or 20 symptoms perfectly overlapped, they would have a dissimilarity of 0/47 or 0%. This measure thus reflects not only which symptoms the two people had in common, but also the common lack of symptoms.

### Mapping of the MHQ to DSM-5 Criteria

MHQ items were first mapped to the symptoms described within the criteria of each of the 10 DSM-5 disorders, based on the closest semantic match (**Figure 3** and Table 2). We note that some disorder criteria included multiple symptoms and therefore mapped to more than one MHQ item. For example, one criterion for depression includes “Diminished ability to think or concentrate, or indecisiveness,” which mapped to MHQ items of *Focus and concentration, Selective attention*, and *Decision-making & risk-taking*. As MHQ questions were formulated from DSM-based assessment tools, all symptoms described in the DSM-5 criteria for these 10 disorders had an MHQ match. The specific criteria rules of the DSM-5 were then applied to arrive at diagnostic rules using the MHQ for each of the 10 disorders (see Table 2).

**Table 2:** Rules for mapping MHQ items to DSM-5 diagnostic criteria.

Depression	Must have at least ONE of: (1) Drive & Motivation OR Curiosity, Interest & Enthusiasm; (2) Feelings of Sadness, Distress or Hopelessness OR Outlook & optimism AND Must have at least FIVE of: (1) Drive & Motivation OR Curiosity, Interest & Enthusiasm; (2) Feelings of Sadness, Distress or Hopelessness OR Outlook & Optimism; (3) Appetite Regulation; (4) Confusion or slowed thinking; (5) Energy Level; (6) Self-worth & Confidence OR Self-image OR Guilt & Blame; (7) Focus & Concentration OR Selective Attention OR Decision-making & Risk-taking; (8) Suicidal thoughts or intentions.
Anxiety	Must have Fear & Anxiety AND Stability & Calmness OR Emotional control AND Must have at least THREE of (1) Restlessness & Hyperactivity; (2) Energy Level; (3) Focus & Concentration OR Selective Attention; (4) Anger & Irritability; (5) Experience of Pain (6) Sleep Quality; (7) Avoidance & Withdrawal
Bipolar	Must have: Mood swings AND Must have at least ONE of: (1) Drive & Motivation OR Curiosity, Interest & Enthusiasm; (2) Feelings of Sadness, Distress or Hopelessness OR Outlook & optimism AND Must have at least FIVE of: (1) Drive & Motivation OR Curiosity, Interest & Enthusiasm; (2) Feelings of Sadness, Distress or Hopelessness OR Outlook & Optimism; (3) Appetite Regulation; (4) Confusion or slowed thinking; (5) Energy Level; (6) Self-worth & Confidence OR Self-image OR Guilt & Blame; (7) Focus & Concentration OR Selective Attention OR Decision-making & Risk-taking; (8) Suicidal thoughts or intentions.
PTSD	Must have a life trauma (or prefer not to say) response on the * < life_trauma>* question (i.e., any response except “I HAVE NOT EXPERIENCED ANY TRAUMA”) AND Must have at least ONE of: (1) Traumatic Flashbacks (2) Nightmares (3) Unwanted, Strange or Obsessive thoughts AND Must have Avoidance & withdrawal AND Must have at least TWO of: (1) Memory (2) Self-worth & Confidence OR Self-image OR Outlook & Optimism; (3) Guilt & Blame; (4) Feelings of Sadness, Distress & Hopelessness; (5) Curiosity, Interest & Enthusiasm OR Drive & Motivation; (6) Relationships with others. AND Must have at least TWO of:(1) Aggression toward others OR Anger & Irritability; (2) Decision-making & Risk-taking; (3) Fear & Anxiety; (4) Focus & Concentration OR Selective Attention; (5) Sleep Quality
OCD	Must have Unwanted, Strange or Obsessive Thoughts AND Repetitive or compulsive actions AND Fear & Anxiety AND Must have at least ONE of: (1) Stability & Calmness; (2) Self-control & Impulsivity; (3) Emotional Control
Schizophrenia	Must have Unwanted, Strange or Obsessive Thoughts AND Hallucinations AND Must have at least ONE of (1) Speech & Language; (2) Repetitive or compulsive actions (3) Drive & Motivation OR Relationships with others OR Social Interactions and Cooperation OR Curiosity, Interest & Enthusiasm
Eating disorder	Must have Appetite Regulation AND Fear & Anxiety AND Self-image
Addiction	Must have Addictions AND Must have at least two of (1) Decision-making & Risk-taking; (2) Emotional Control (3) Avoidance & Withdrawal (4) Relationships with others; (5) Self-control & Impulsivity
ADHD	Must have at least FOUR of (1) Focus & Concentration; (2) Selective Attention; (3) Drive & Motivation; (4) Planning and Organization; (5) Memory OR Must have Restlessness & Hyperactivity AND Self-control & Impulsivity
ASD	Must have at least THREE of: (1) Social Interactions and Cooperation; (2) Relationships with others; (3) Repetitive or compulsive actions; (4) Adaptability to Change; (5) Sensory sensitivity; (6) Selective Attention OR Focus & Concentration OR Curiosity, Interest & Enthusiasm; (7) Empathy

*Each item needed to be above the rating threshold of ≥8 for problem items or ≤ 1 for spectrum items to be considered a clinically significant symptom*.

For example, within the DSM-5, a positive diagnosis for depression requires that the individual has experienced at least 5 out of a list of 8 different criteria with one of them being a depressed mood or loss of interest or pleasure. These criteria, when applied to the MHQ elements, required that the symptom profile (MHQ elements meeting the symptom threshold) included at least *Feelings of Sadness, Distress or Hopelessness* or poor *Outlook & Optimism* AND/OR poor *Drive & Motivation* or poor *Curiosity, Interest & Enthusiasm*, AND must have least 5 of (1) *Feelings of Sadness, Distress or Hopelessness* or poor *Outlook & Optimism;* (2) poor *Drive & Motivation* or poor *Curiosity, Interest & Enthusiasm;* (3) poor *Appetite Regulation;* (4) *Confusion or slowed thinking;* (5) low *Energy Level;* (6) low *Self-image* or low *Self-worth & Confidence* or *Guilt & Blame;* (7) poor *Decision-making & Risk-taking* or poor *Focus & Concentration* or poor *Selective Attention;* (8) *Suicidal thoughts or intentions* (**Figure 3** and Table 2).

Although this MHQ diagnostic criteria mapping does not mean that the individual would be diagnosed with that disorder in the context of a clinical interview, it indicates that their pattern of clinical symptoms broadly aligned with the diagnostic criteria for that disorder. However, we note the caveat that for bipolar disorder, symptoms denoted extreme versions of positive assets (e.g., grandiosity and decreased need for sleep) that were not fully articulated within the MHQ, while for OCD the MHQ items were broader (e.g., obsessive thoughts were incorporated within a general item reflecting *Strange, unwanted and obsessive thoughts;* see section Limitations). Furthermore, specific criteria relating to symptom timing were not included, as this is not included in the MHQ which assesses the individual's current perception of themselves.

### Within Disorder Analysis

To determine the heterogeneity of symptom profiles within a disorder group (i.e., all those who mapped to a particular disorder as described in section Mapping of the MHQ to DSM-5 Criteria), we computed the symptom dissimilarity (as defined in section Computing Dissimilarity of Symptom Profiles) between all pairs of individuals within the group and determined the average symptom dissimilarity and the standard deviation (SD) of the dissimilarity between all pairs. We similarly computed the dissimilarity of symptom profiles of each individual within a disorder group to each individual in a random demographically matched group, computing statistics on these distributions as described in section Statistics. We note that 10 iterations of random groupings (each *N* = 3,333) yielded similar results (average symptom dissimilarity across all pairs of individuals ± SD was 40.5 ± 11.3% and ranged from 39.5 to 41.2%) indicating that any comparison would provide a similar result.

### Between Disorder Analysis

We next compared the symptom profiles of individuals between two disorder groups. To do so, we first computed the symptom dissimilarity between each individual from one disorder group and each individual from another disorder group. We did so by including all individuals in each group (but excluding self-comparisons), and also by removing those individuals who were part of both disorder groups (i.e., would be considered “comorbid”). This computation was performed across the whole sample, and separately across different country, age and gender demographic groups.

### Statistics

To determine whether individuals within a disorder group were statistically more homogenous than a randomly selected group of individuals with at least one disorder, we computed the *p*-value of the difference in the distribution of dissimilarities for each disorder group compared to a randomly selected group using a two tailed *t*-test.

To test the hypothesis that each disorder group represented sufficient symptom homogeneity such that it represented a cluster that could be differentiated from across the disorder space, we used the Hopkins Statistic (H) as described in (90). This statistic compares the distance (i.e., symptom dissimilarity) of elements from each disorder group to the nearest neighbor within the disorder group and to the nearest neighbor in the randomly selected group as follows:


$$\begin{array}{l} \text{H}=\frac{\overset{\text{n}}{\underset{\text{i}=1}{\sum }}{\text{d}}_{\text{i}}}{\overset{\text{n}}{\underset{\text{i}=1}{\sum }}{\text{d}}_{\text{i}}+\overset{\text{n}}{\underset{\text{i}=1}{\sum }}{\text{r}}_{\text{i}}} \end{array}$$

Where *d* is the nearest neighbor distance within the disorder group and *r* is the nearest neighbor distance to the random group.

If the total nearest neighbor distance between symptom profiles within the disorder group is, on average, the same as the distance to the nearest neighbor from the random group, H should be about 0.5, implying that the disorder group does not represent a cluster that can be differentiated from random. On the other hand, if the disorder groups each represent relatively compact and isolated clusters, H should be larger than 0.5 and almost equal to 1.0 for very well-defined clusters.

## Results

### Prevalence and Profile of Clinical Symptoms

**Figure 2:**
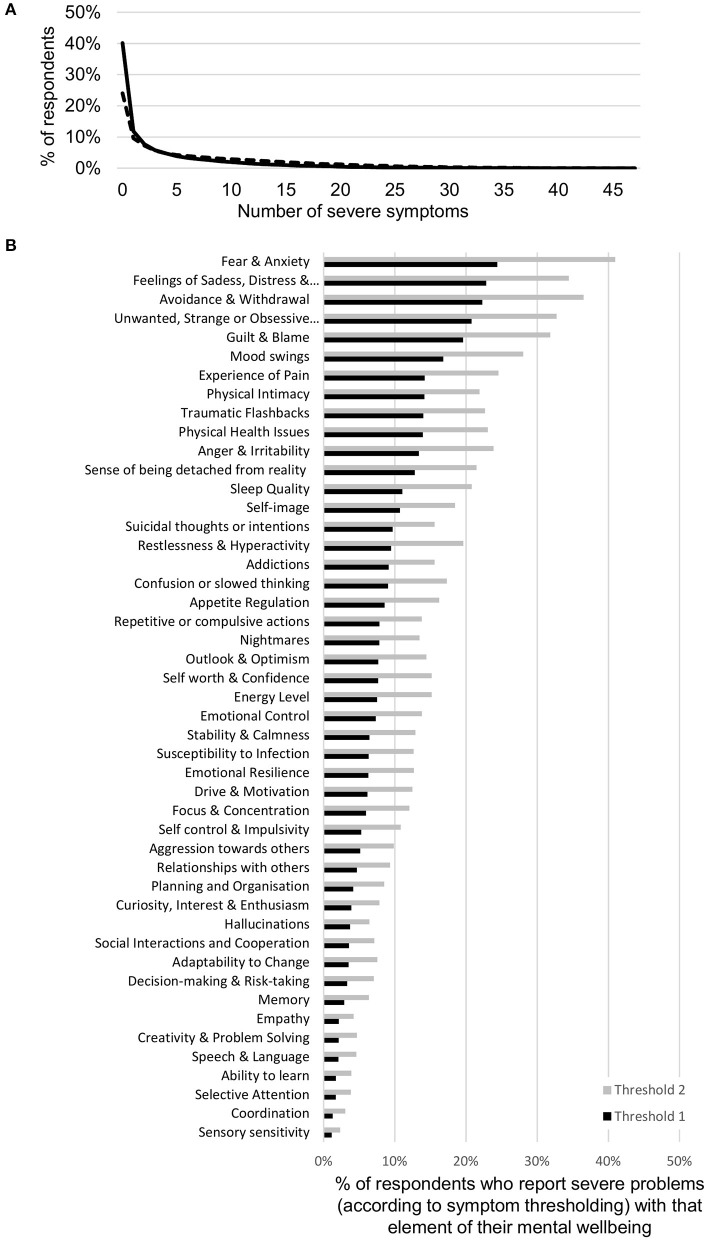
Symptom prevalence in the sample. **(A)** Prevalence of number of clinical mental health symptoms in the sample as defined by a threshold of severity in the MHQ (≥8 for problem items and ≤1 for spectrum items; black line). Dotted line denotes the prevalence when the threshold is shifted by one point (≥7 for problem items and ≤2 for spectrum items). The percentage with each higher number of clinical symptoms decreases exponentially. **(B)** The proportion of respondents who reported severe problems with each of the 47 elements of mental well-being included in the MHQ, as defined by a threshold of severity in the MHQ (≥8 for problem items and ≤1 for spectrum items; black bars). Prevalence of specific symptoms across the sample ranged from 24.4 to 1.1%. Gray bars denote the proportion when the threshold is shifted by one point (≥7 for problem items and ≤2 for spectrum items).

The prevalence of clinical symptoms in the sample was dependent on the threshold used to determine clinical significance. While the DSM-5 typically specifies that a mental aspect should be considered a symptom if it occurs “nearly every day,” “persistently,” or “more days than not,” in reality, thresholding is determined using a wide variety of criteria across diagnostic screening tools, from severity and frequency to timing and duration of symptoms (35). Here the criterion used was severity of impact on one's ability to function. At the stringent threshold criterion used to define a clinical symptom throughout this paper (see Symptom Criteria section in the Methods), 40.1% of all respondents across the sample had no clinical symptoms, while 25.9% had more than 5 clinical symptoms (Figure 2A). However, when the threshold was shifted one point (dotted line in 2A; ≥7 for problem items; ≤2 for spectrum items), it resulted in only 24.0% of all respondents having no clinical symptoms while 44.4% had more than 5. The fraction of the sample with successively larger numbers of clinical symptoms decreased in a manner that was best fit by an exponential function. Thus, purely from the perspective of number of clinical symptoms, there was no specific distinction where one might draw the line between normal and disordered. Changing the threshold to define a clinical symptom less stringently in terms of severity decreased the fraction of people with *no* clinical symptoms but did not change the shape of this curve (Figure 2A dotted line). Within this distribution, some clinical symptoms were more prevalent than others (Figure 2B; black bars). Four clinical symptoms were reported by >20% of the sample including *Unwanted, strange or obsessive thoughts, Feelings of sadness, distress or hopelessness, Fear and anxiety* and *Avoidance and withdrawal*. Others such as problems with *Sensory sensitivity, Coordination* and *Selective attention* were rarer, with rates <2%. When the threshold was shifted one point (gray bars in 2B; ≥7 for problem items; ≤ 2 for spectrum items), the prevalence of different symptoms increased across all elements, but to a greater or lesser degree across elements. We note that while there were significant differences in symptom prevalence between age and gender groups, the exponential structure of symptom prevalence was the same. While not shown here, differences between age and gender groups have been shown with a subset of this data previously (91). Given these differences, our analysis looked at the whole sample as well as across different demographic groups.

### Mapping of Clinical Symptoms to the DSM-5 Criteria

**Figure 3:**
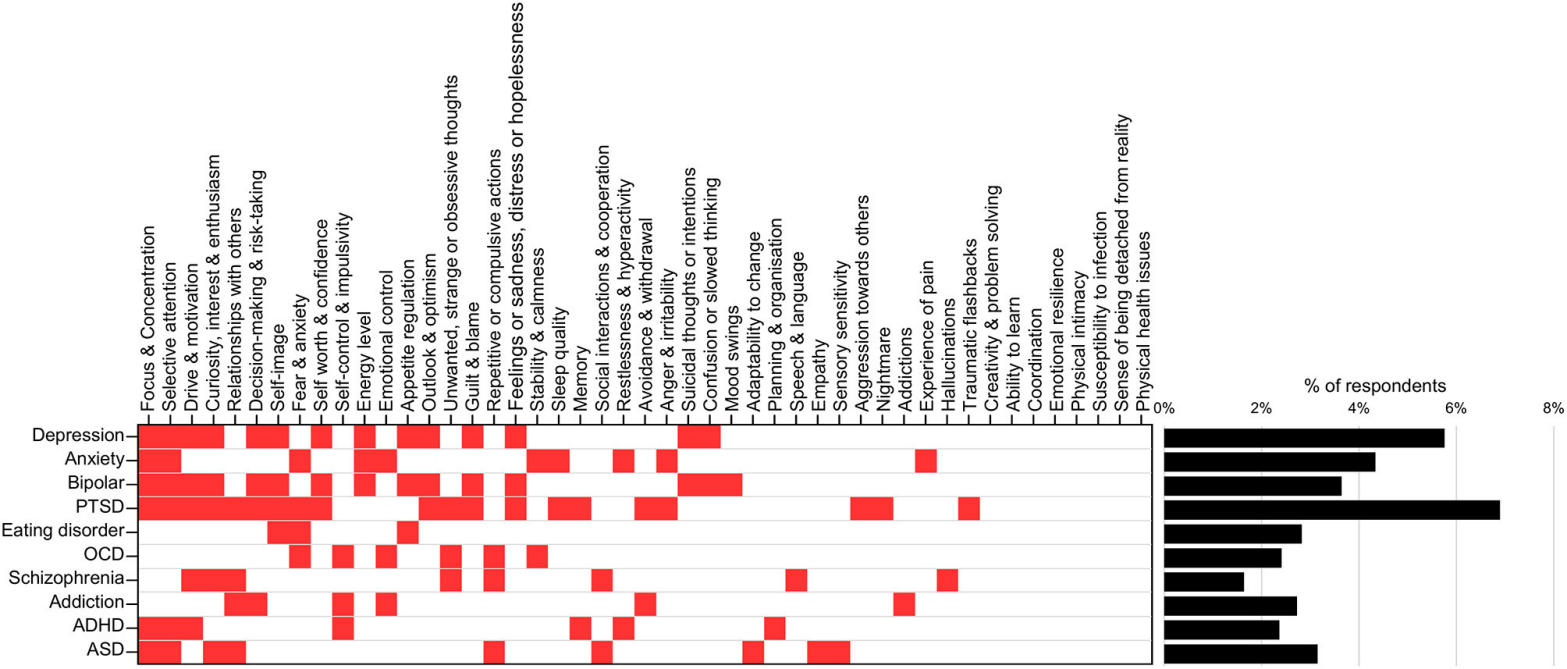
Mapping of MHQ symptoms to DSM-diagnostic criteria. MHQ items corresponding to each DSM-5 defined disorder (left; red squares) and associated prevalence of each disorder following application of diagnostic rules (right). Full mapping and rules shown in Table 2.

Figure 3 (left) shows the mapping of elements or items of the MHQ to each of 10 mental health disorders, based on the DSM-5 diagnostic criteria. Only 12 out of 47 MHQ items that mapped to DSM-5 disorder criteria were unique to one “disorder” label. However, the specific criteria for each disorder differed (see Table 2). Applying these rules to the clinical symptoms of each individual (as determined in Symptom Criteria section in the Methods), the prevalence for each of these disorders ranged between 1.6% (schizophrenia) to 6.9% (PTSD) (Figure 3, right). These fall within the broad ranges of prevalence across epidemiological studies. For example, the prevalence of ADHD within the current adult sample (2.4%) closely matched the estimated global prevalence of ADHD of 2.8% (92). Similarly, the prevalence of depression within this study (5.8%), lay within the range of prevalence estimated by some studies (3, 93, 94), although is lower than more recent estimates from other sources (95, 96). However, prevalence estimates vary substantially depending on the assessment tool used, the geographical location, and also the timing in relation to the Covid-19 pandemic (7, 96–103). For example, the epidemiological estimates for PTSD varied between 7 and 53.8% in one recent meta-analysis (7).

### DSM-Diagnostic Comorbidity Profiles

**Figure 4:**
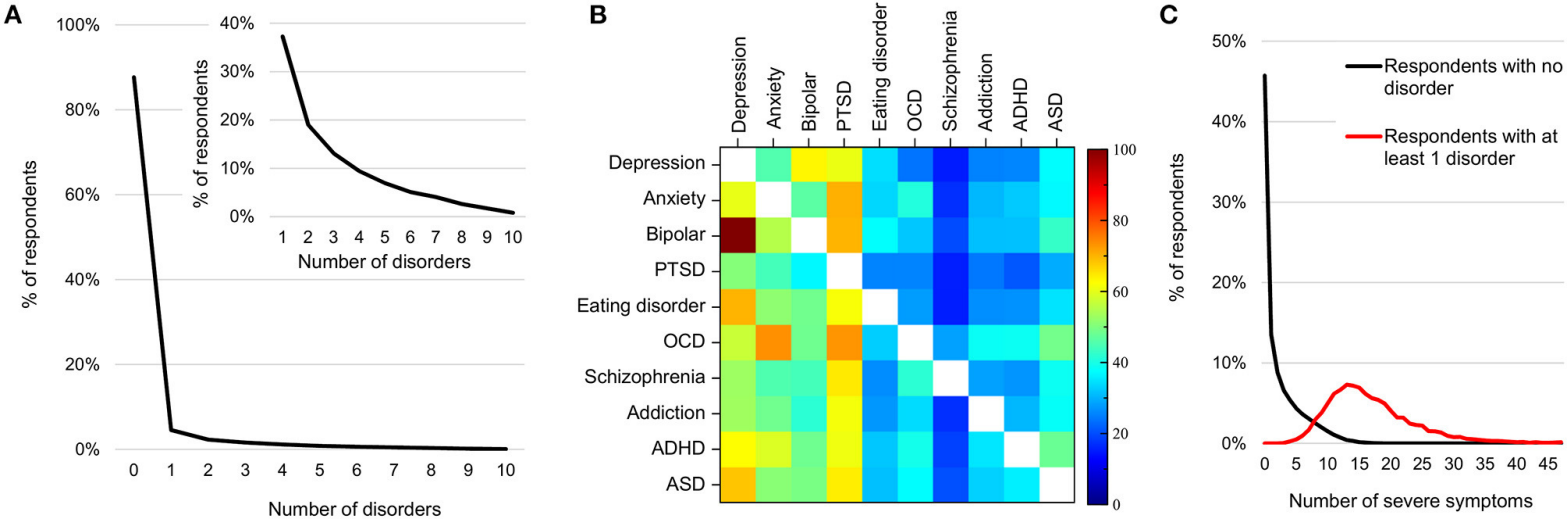
Prevalence and comorbidity among DSM-disorders. **(A)** Percentage of individuals by number of disorders to which they map. Inset shows the mapping of the 12.3% with one or more disorder. **(B)** Percentage of individuals with each disorder who also map to each other disorder (numbers in Supplementary Table 3). **(C)** Distribution of number of clinical symptoms across individuals who do not map to any disorder criteria (black line) and those who map to at least one (red line).

Overall, 12.3% of all respondents mapped to one or more disorders (Figure 4A), within which 62.7% mapped to more than one disorder (Figure 4A, inset) in line with findings that comorbidity is the norm (33, 34, 71). Figure 4B shows comorbidity for the 10 disorders by rows (numbers in Supplementary Table 3). For example, row 1 represents the fraction of individuals whose symptom profiles aligned with the criteria for depression and who also aligned with the criteria for each of the other disorders (shown across the columns). Here 25.7 and 37.3% of those with depression also met the criteria for ADHD and ASD, respectively. Conversely, 62.6 and 68.1% of individuals who mapped to ADHD and ASD criteria, respectively, also aligned with criteria for depression. Note from prevalence estimates in Figure 3, while 5.8% of individuals met the criteria for depression, only 2.4 and 3.2% met the criteria for ADHD and ASD, respectively.

### Symptom Prevalence Beyond DSM-Disorder Criteria

The minimum number of clinical symptoms required for a diagnosis of each of these 10 disorders, as per the DSM-5, ranges from 3 to 6. For instance, depression requires a minimum of 5 symptoms across 5 criteria out of a possible 18 symptoms and 8 criteria. Nonetheless, among those who mapped to at least one disorder, the average number of clinical symptoms was 20 (median 15), substantially higher than the minimum criteria for any one disorder (Figure 4C; red curve). On the other hand, 58.7% of respondents with 5 or more clinical symptoms did not map to any of the 10 disorder criteria (Figure 4C, black curve).

### Symptom Heterogeneity Within Disorders

If a disorder group had significantly lower symptom dissimilarity, i.e., more homogeneous symptom profiles, compared to a randomly selected group, this would indicate that the DSM-5 diagnostic criteria for that disorder was good at separating individuals by a particular profile. We therefore first evaluated the heterogeneity of symptom profiles within each DSM-5 disorder group to see how they compared to the heterogeneity of groups of randomly selection individuals from across any of the 10 disorder groups. We hypothesized that overall, the symptom profiles of individuals within each DSM-5 disorder group would be more homogeneous than a randomly selected group and therefore reasonably well-separated.

**Figure 5:**
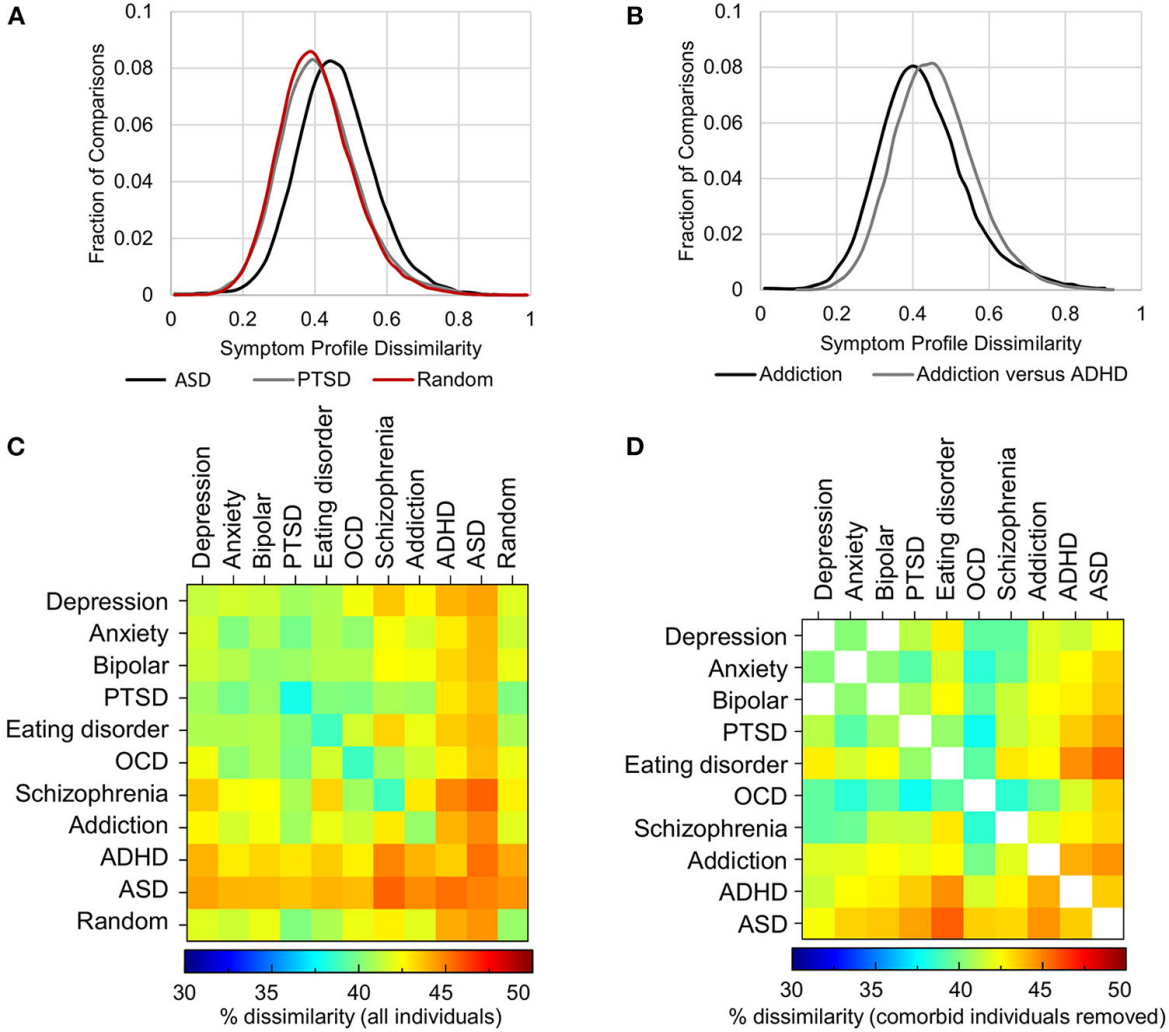
Heterogeneity of symptom profiles within and between disorders. **(A)** Distribution of symptom profile dissimilarity across all individuals who map to criteria for PTSD (gray), ASD (black) and a randomly selected groups of individuals from the pool of individuals with at least one of the 10 disorders (red). **(B)** Distribution of the dissimilarity of symptom profiles of all individuals mapping to the diagnostic criteria for addiction (black) and the dissimilarity of symptom profiles between those with addiction and those with ADHD excluding comorbid individuals. **(C)** Symptom dissimilarity within and between each pair of disorders ranges from 37.9% (within PTSD) to 45.6% (between ASD and Schizophrenia). **(D)** Symptom dissimilarity as in **(C)** but with comorbid individuals excluded ranged from 37.8 to 45.7%.

First, within each disorder, the heterogeneity (average dissimilarity ± SD) ranged from 37.9 ± 10.7 for PTSD (i.e., the most consistent) to 44.9 ± 10.1 for ASD (the most heterogeneous), while the mean heterogeneity for randomly selected groups was 40.5 ± 11.3% (Figure 5A, diagonal of Figure 5C and Table 3). Only PTSD (which had the highest prevalence in the sample, see Figure 3) had more than 5% lower average dissimilarity (6.4%) and therefore greater homogeneity than the average of the random groups, while ADHD and ASD had more than 5% higher average dissimilarity and therefore greater heterogeneity than the average of the random groups (7.2 and 10.9%, respectively; Figures 5A,C and Table 3). While the differences in dissimilarity values were small, even very small differences were statistically significant by *t*-test (all *p* < 0.0009), since the sample size was large.

**Table 3:** Dissimilarity of symptom profiles within each disorder group and the comparison against symptom profiles within groups of randomly selected individuals from any disorder group.

	**Dissimilarity within disorder group (average ± SD)**	**Dissimilarity between disorder group and random group (average ± SD)**	***T*-test**	**H-statistic**
Depression	41.3 ± 9.8%	41.9 ± 10.2%	*P* < 0.00000	0.51
Anxiety	40.1 ± 10.0%	41.5 ± 10.6%	*P* = 0.00091	0.50
Bipolar	40.4 ± 9.4%	42.1 ± 10.6%	*P* < 0.00000	0.50
OCD	38.7 ± 10.7%	42.1 ± 11.4%	*P* < 0.00000	0.50
Schizophrenia	38.8 ± 12.7%	42.7 ± 11.8%	*P* < 0.00000	0.50
PTSD	37.9 ± 10.7%	40.0 ± 11.4%	*P* < 0.00000	0.50
Eating disorder	38.8 ± 11.4%	40.9 ± 11.4%	*P* < 0.00000	0.49
Addiction	40.5 ± 10.9%	41.9 ± 11.0%	*P* < 0.00000	0.50
ADHD	43.4 ± 11.9%	44.1 ± 11.7%	*P* < 0.00000	0.52
ASD	44.9 ± 10.1%	44.6 ± 10.4%	*P* < 0.00000	0.53
All random groups	40.5 ± 11.3%	N/a	N/a	N/a

Given that the difference in heterogeneity from the random groups was significant, although in both positive and negative directions (i.e., more and less heterogeneous), we also looked at the Hopkins Statistic, a measure of how well disorder groups could be separated. The Hopkins Statistic measures how much the distance (dissimilarity) between nearest neighbors of elements in the disorder group differs from the distance to the nearest neighbor in the random group such that a value of 0.5 would indicate that there was no difference. On the other hand, separable groups where the elements have closer neighbors within their group relative to a random group would typically have a Hopkins Statistic value >0.7 (see section Methods). Here the Hopkins statistic ranged from 0.49 to 0.53 across all 10 disorders indicating that they were not distinguishable from a random sample and therefore did not represent a valid cluster.

We similarly ran this analysis for different demographic segments in order to determine whether there was any difference for a particular demographic (e.g., age, gender, country) such that the DSM-5 criteria better separated individuals in one particular group. However, the results were similar irrespective of demographic (Supplementary Table 4).

### Symptom Dissimilarity Between Disorders

As randomly selected groups would typically have a composition that reflected the prevalence of disorders and their comorbidities, we next hypothesized that the symptom profiles between disorders might be significantly different from one another when comorbid individuals were removed. Significantly, *between* disorders, dissimilarity was not much higher than the within disorder dissimilarity, ranging from 39.8% (between Anxiety and PTSD) to 45.6% (between Schizophrenia and ASD) (Figure 5C and Table 4). In addition, contrary to expectation, excluding all comorbid individuals from the comparison *decreased* heterogeneity, although not substantially, as comorbid individuals tended to have a larger number of symptoms overall, and therefore greater symptom dissimilarity (Figure 5D and Table 4). Only in 18% of comparisons did heterogeneity increase. Overall, in either direction the maximal difference was 5.7%. Figure 5B shows an example of the within disorder heterogeneity for addiction compared to the heterogeneity between individuals with addiction and ADHD after removing those individuals who were comorbid for both. This demonstrates the very small difference in dissimilarity among those with addiction compared to the dissimilarity between those who had addiction and those who had ADHD (but not both).

**Table 4:** Dissimilarity of symptom profiles between each disorder group, with and without comorbid individuals removed.

	**Dissimilarity between disorder group (average ± SD)**	**Dissimilarity between disorder group; comorbid individuals removed (average ± SD)**
Depression & Anxiety	41.6 ± 9.6%	40.2 ± 9.0%
Depression & Bipolar	41.4 ± 9.7%	N/a
Depression & OCD	42.3 ± 10.0%	41.2 ± 9.2%
Depression & Schizophrenia	43.6 ± 10.6%	42.8 ± 10.2%
Depression & PTSD	40.7 ± 10.0%	39.4 ± 9.9%
Depression & Eating Disorder	40.9 ± 10.3%	39.4 ± 9.7%
Depression & Addiction	42.7 ± 9.9%	41.9 ± 9.6%
Depression & ADHD	44.0 ± 10.1%	41.5 ± 8.6%
Depression & ASD	44.3 ± 9.6%	42.4 ± 8.3%
Anxiety & Bipolar	41.0 ± 9.5%	40.3 ± 8.9%
Anxiety & OCD	40.4 ± 10.2%	39.3 ± 9.4%
Anxiety & Schizophrenia	42.3 ± 10.7%	41.7 ± 10.2%
Anxiety & PTSD	39.8 ± 10.2%	38.3 ± 9.8%
Anxiety & Eating Disorder	41.0 ± 10.2%	39.6 ± 9.2%
Anxiety & Addiction	41.7 ± 10.2%	41.9 ± 9.9%
Anxiety & ADHD	42.9 ± 10.6%	42.5 ± 10.0%
Anxiety & ASD	43.9 ± 9.7%	43.4 ± 8.9%
Bipolar & OCD	41.1 ± 9.7%	40.8 ± 9.1%
Bipolar & Schizophrenia	42.5 ± 10.3%	42.5 ± 10.0%
Bipolar & PTSD	40.5 ± 9.9%	39.5 ± 9.4%
Bipolar & Eating Disorder	41.0 ± 10.0%	41.5 ± 9.5%
Bipolar & Addiction	42.3 ± 9.9%	42.5 ± 9.5%
Bipolar & ADHD	43.3 ± 10.0%	42.8 ± 9.2%
Bipolar & ASD	43.9 ± 9.5%	43.6 ± 8.7%
OCD & Schizophrenia	40.7 ± 11.2%	40.4 ± 10.6%
OCD & PTSD	39.9 ± 10.7%	37.8 ± 10.1%
OCD & Eating Disorder	41.6 ± 10.5%	41.4 ± 9.4%
OCD & Addiction	41.5 ± 10.7%	42.1 ± 10.1%
OCD & ADHD	42.7 ± 11.2%	43.5 ± 10.3%
OCD & ASD	43.8 ± 10.3%	44.3 ± 9.7%
Schizophrenia & PTSD	40.9 ± 11.4%	39.3 ± 10.8%
Schizophrenia & Eating Disorder	43.4 ± 10.9%	42.9 ± 9.9%
Schizophrenia & Addiction	42.9 ± 11.2%	42.6 ± 10.5%
Schizophrenia & ADHD	44.9 ± 11.8%	44.7 ± 11.1%
Schizophrenia & ASD	45.6 ± 10.9%	45.7 ± 10.5%
PTSD & Eating Disorder	40.1 ± 10.6%	38.4 ± 10.0%
PTSD & Addiction	40.7 ± 10.5%	39.8 ± 10.3%
PTSD & ADHD	43.0 ± 11.0%	41.7 ± 10.7%
PTSD & ASD	43.7 ± 10.0%	43.4 ± 9.9%
Eating Disorder & Addiction	42.1 ± 10.7%	41.9 ± 10.1%
Eating Disorder & ADHD	43.5 ± 10.8%	42.7 ± 9.7%
Eating Disorder & ASD	44.0 ± 10.1%	43.3 ± 9.0%
Addiction & ADHD	43.9 ± 11.2%	44.1 ± 10.5%
Addiction & ASD	44.7 ± 10.3%	44.6 ± 9.8%
ADHD & ASD	45.3 ± 10.4%	43.5 ± 8.6%

**Figure 6:**
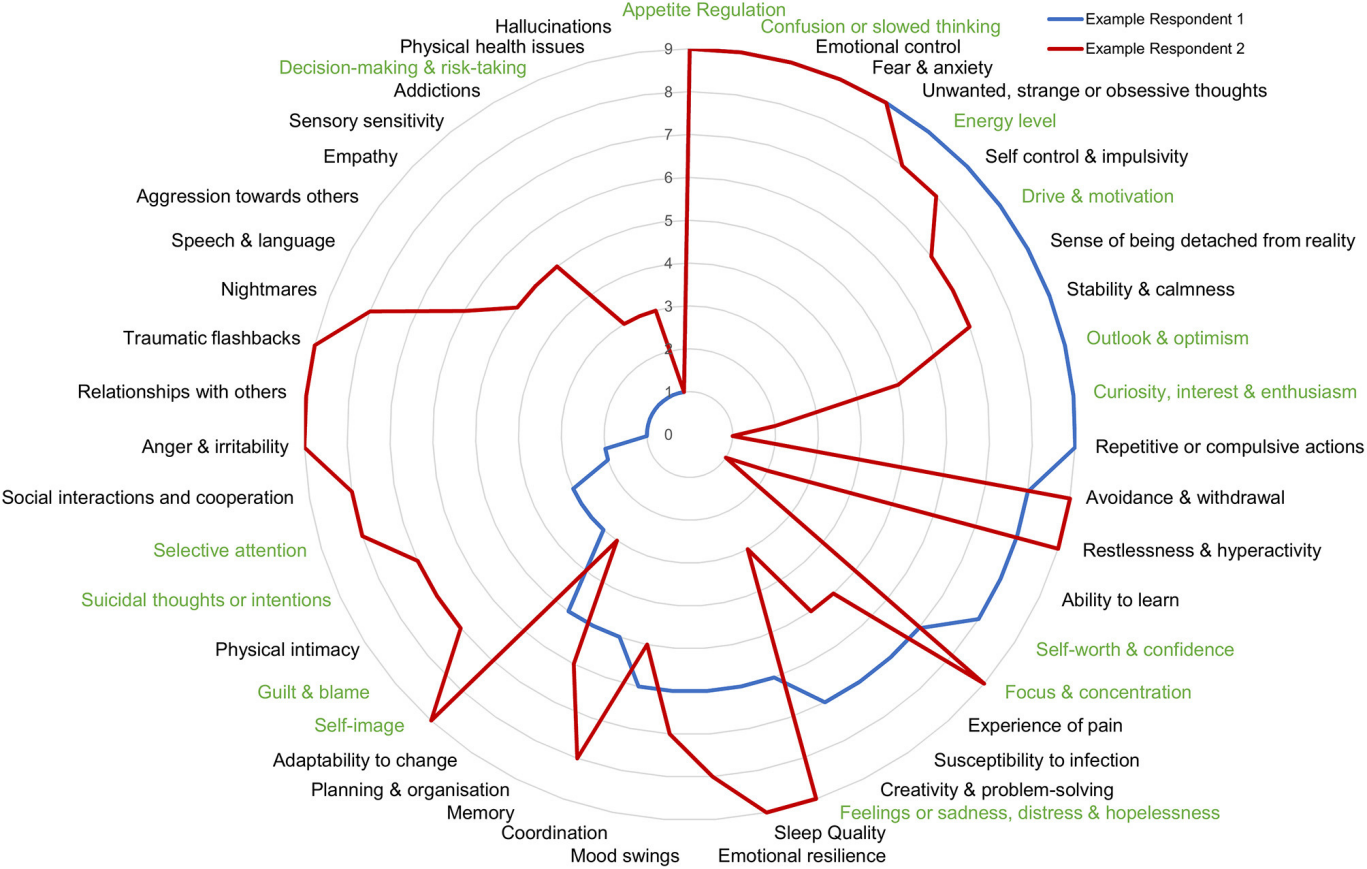
Symptom profile of 2 individuals who map to DSM-criteria for depression. Comparison of symptom profiles of 2 individuals mapping to diagnostic criteria for depression is an example of the within disorder symptom heterogeneity. Higher numbers toward the outside of the circle represent a greater problem.

Thus, contrary to expectation, the DSM-5 criteria by which disorders are defined does not separate individuals based on their overall symptom profile and is closely equivalent to a random drawing of individuals. As a demonstration of the heterogeneity within disorders, we show the clinical symptom profile of 2 individuals who met the diagnostic criteria for depression (Figure 6). If these individuals were only considered on the basis of a depression screening, their dramatically different symptom profiles would be missed.

## Discussion

The heterogeneity of symptom profiles within disorders, and comorbidity across disorders, are known to present a significant challenge to the effective diagnosis, treatment and research of mental illness (20, 23, 31). Here we quantify the degree of heterogeneity of comprehensive symptom profiles of 107,349 individuals within and across DSM-diagnostic criteria. While 58.7% of those with 5 or more clinically significant symptoms did not map to the diagnostic criteria of any of the 10 DSM-5 disorders, those with symptom profiles that mapped to at least one disorder reported, on average, 20 clinically significant symptoms. The heterogeneity of symptom profiles was almost as high within a disorder group (average dissimilarity of 40.5% across all disorders) as between 2 disorder groups (41.8% on average with comorbid individuals removed) and no individual disorder group was separable from randomly selected groups of individuals with at least one disorder, with the Hopkins statistic ranging between 0.49 and 0.53. Overall, the DSM-5 disorder criteria failed to separate individuals by symptom profiles any better than random assignment. Thus, rather than representing a method of separating groups based on symptoms, disorder labels primarily serve to emphasize a particular subset of symptoms without consideration of the entire symptom profile. Given that the symptom criteria of the DSM-5 do not specifically reflect any known underlying biology or etiology, this calls into question their utility as a diagnostic system and likely plays a greater role in hindering than helping progress in the understanding and treatment of mental health challenges. We discuss here the implications of these results for how we consider mental health epidemiology, clinical diagnosis, treatment, and research.

### Estimating Prevalence and Mental Health Epidemiology

Across the sample, symptom prevalence decreased systematically with an exponential decay. Thus, drawing the line between how many severe symptoms are normal vs. clinical is essentially an arbitrary judgement. Across the sample, using the more stringent threshold of negative life impact to define clinical significance, 12.3% had symptom profiles that aligned with at least one DSM-5 disorder diagnosis. However, changing the threshold of clinical significance within the MHQ rating framework by one severity point changed clinical prevalence to 25.8%. While prevalence estimates in this data, based on point estimates and using the most stringent threshold criteria, matched up with some epidemiological prevalence estimates (3, 92–94), studies have wide ranges that depend on the tool used, the thresholds considered, as well as geography and time period (7, 96–103). For example, a recent meta-analysis found that point prevalence of depression ranged from 3.1 to 87.3% across 48 studies and 7 populations (97). Notwithstanding the heterogeneity of disorders, this highlights that there is no absolute epidemiological estimate but rather that each estimate must be qualified by the thresholds used, as well as other factors. Thus, changes in prevalence across time periods cannot be performed using different tools. It also raises the question of who decides the appropriate threshold, or boundary, between a disorder and the normal variation of human existence (104–106). This has implications for numerous societal aspects, such as the threshold at which a person warrants medical attention as well as debate over financial and resource allocation.

### Implications for Clinical Diagnosis and Research

These results highlight the quantitative extent of the challenge of utilizing DSM-5 based criteria for diagnosis and treatment selection, where symptoms of those who map to DSM-5 criteria are as heterogeneous within disorder labels as between and cannot be differentiated from random. Given this heterogeneity, it is no surprise that only 39% of clinicians report often or always referring to written DSM criteria during an initial evaluation and approximately a third often or always use not otherwise specified (NOS) categories, primarily due to the patient not meeting criteria for a specific category (107). These results also illustrate the challenges of selecting patients or participants into a research study based on their meeting criteria for a disorder label. When participants are recruited into a trial based on these diagnostic criteria, they are as symptomatically heterogeneous a pool as if one were to simply recruit randomly among those with mental health distress from any of these 10 disorders. This likely contributes substantially to the challenge of obtaining clear results in clinical trials where outcomes are rarely unequivocal and typically successful for a low proportion of patients (15). In addition, efficacy of a treatment with respect to the subset of symptoms emphasized by a DSM-5 based disorder label may not reflect overall efficacy in treating mental well-being, as other symptoms that are not considered in a particular diagnosis may fail to improve or even get worse.

### Limitations

It is important that we acknowledge some limitations of this data and study. First, symptom profiles are obtained through a self-report questionnaire. While the assessment has now been extensively tested for reliability and validity (89), this would nonetheless limit the inclusion of individuals who are so impaired that they may be unable to take an online survey, or unable to understand the questions sufficiently to answer them.

Second, the sample consisted of self-selected individuals whose symptom profiles may differ from those who chose not to take an assessment. However, this concern is mitigated by the large sample size, broad demographic representation and similar results obtained across demographics. Even if the results only apply to those who would present for mental health assessment this is nonetheless important as they represent the population more likely to be seeking help.

Third, the sample was acquired during the Covid-19 pandemic during which symptom profiles may differ from pre-pandemic times. Should this be the case, it would highlight the challenge of a hard and fast diagnostic system that does not consider the evolution of mental health challenges over time.

Fourth, the criteria mapping process was limited by the absence of specific information pertaining to symptom frequency within the MHQ for each item. The threshold of 8 was determined as an appropriate selection for at least one problem item (*Feelings of sadness, distress or hopelessness*), equivalent on average to experiencing the symptom 5 days per week in alignment with the DSM-5 requirement of experiencing the symptom “nearly every day” for depression. However, it is possible that the correspondence between frequency and life impact rating may differ from item to item and so other threshold values may have been more appropriate for other items. Nonetheless, while one would expect that most thresholds would not be far off the mark, this highlights the challenges of a threshold-based approach which plagues most, if not all, assessment tools.

Finally, the presence of broad or imperfect matches for certain symptoms pertaining to OCD and bipolar disorder could have affected the accuracy of the mapping and the specific values of prevalence and overlap as derived from this data. While this must be verified in future studies, the inaccurate mapping of one or two symptoms is not likely to impact the overall finding—that overall symptom profiles are as heterogeneous within disorder groups as between and not separable as a group from randomly selected individuals with any disorder. This is because the DSM-5 typically considers only between 3 and 6 symptoms for a diagnosis whereas individuals present with 20 symptoms on average (Figure 4C). Therefore, while more accurate mapping may improve the symptom overlap by one or two symptoms, this would not move the needle substantially since the 15–17 other symptoms that the diagnosis did not consider would contribute far more to the heterogeneity.

Nonetheless, it is important to compare the MHQ criteria mapping for diagnostic determination to more commonly used assessments, for example mapping against the PHQ-9 (74) and the GAD-7 (75) to determine the alignment between MHQ criteria for depression and anxiety, and the scores from these two questionnaires, respectively. Furthermore, validation of these results with an alternative questionnaire where questions are phrased differently but still cover all symptoms comprehensively would also be important.

### Future Directions

Despite the many challenges of the DSM-5, there is no denying that it is deeply embedded in the fabric of clinical, economic and social decision-making. Indeed 64% of clinicians often or always use the diagnostic codes for administrative or billing purposes and 55% find it very or extremely useful for communicating clinical diagnoses with colleagues and other healthcare professionals (107). However, given the broad reaching negative implications of a disorder classification system that is indistinguishable from random groupings, it is exceedingly important to identify and develop alternative approaches.

From the symptom perspective, we advocate for an approach that is rooted in empirical understanding of symptoms clusters. A stepwise change toward disorder agnostic phenotypes of symptom profiles would have multiple benefits. First, it would allow clinicians to obtain a complete picture of patient symptoms so that they could make more informed treatment decisions based on the whole patient experience leading to more streamlined or personalized treatment pathways. Second, it would further support the application of transdiagnostic frameworks such as RDoC (65). Third, it would aid the search for underlying etiologies, and the identification of social determinants, by allowing phenotypic testing of the efficacy of medications and interventions. In the quest to construct empirical phenotypes of symptoms, different mechanisms of constructing and comparing symptom profiles should be explored. For example, dissimilarity is likely amplified by thresholding, and comparisons that look across the scale of impact may yield a clearer picture.

Finally, it would also be important to utilize large-scale data of comprehensive symptom profiles, such as we have used here, to understand the relative stability of symptoms within individuals and the relationships between symptoms. Multiple approaches have already been proposed in this direction. For example, network approaches (49, 50) have been utilized to identify which symptoms are having the greatest impact in sustaining other symptoms, introducing possibilities for specific targeting of symptoms that will likely have the greatest impact on clinical outcomes (56–58). Another approach, HiTOP (67–69), proposes a hierarchical framework that combines individual symptoms into components or traits, assembling them into empirically derived syndromes, and finally grouping them into psychopathology spectra (e.g., internalizing and externalizing). We note that the MHQ is a symptom profiling tool, rather than a diagnostic framework, and thus would be envisaged as a transdiagnostic or disorder agnostic assessment that could be used to support insights arising from network studies, or those using frameworks such as HiTOP and RDoC. Ultimately, various approaches proposed should be tested to determine which provides the closest correspondence to clinical trial response criteria and insight in relation to physiological parameters.

## Data Availability Statement

The datasets presented in this study can be found in online repositories. The names of the repository/repositories and accession number(s) can be found at: https://sapienlabs.org/mhm-data-access-request/.

## Ethics Statement

The studies involving human participants were reviewed and approved by Health Media Lab Institutional Review Board. Participation was anonymous and involved online informed consent.

## Author Contributions

JN and TT conceptualized, designed, led the study, interpreted the data, drafted the manuscript, and made critical revisions. JN and VP performed the analysis. JN created the figures. JN, TT, and VP approved the final version and agreed to be accountable for all aspects of the work. All authors contributed to the article and approved the submitted version.

## Funding

This work was supported by Sapien Labs using private unrestricted donor funding. Sapien Labs was a 501(c)(3) not for profit research organization founded in 2016 with a mission to understand and enable the human mind.

## Conflict of Interest

TT received a grant award from the National Institute of Mental Health (NIMH) to develop a commercial version of the MHQ tool referenced herein. The remaining authors declare that the research was conducted in the absence of any commercial or financial relationships that could be construed as a potential conflict of interest.

## Publisher's Note

All claims expressed in this article are solely those of the authors and do not necessarily represent those of their affiliated organizations, or those of the publisher, the editors and the reviewers. Any product that may be evaluated in this article, or claim that may be made by its manufacturer, is not guaranteed or endorsed by the publisher.
