# Supplementary figures and images for: Poor Separation of Clinical Symptom Profiles by DSM-5 Disorder Criteria
Source: Front Psychiatry. 2021 Nov 29;12:775762. doi: 10.3389/fpsyt.2021.775762 (PMC8669440; doi:10.3389/fpsyt.2021.775762)

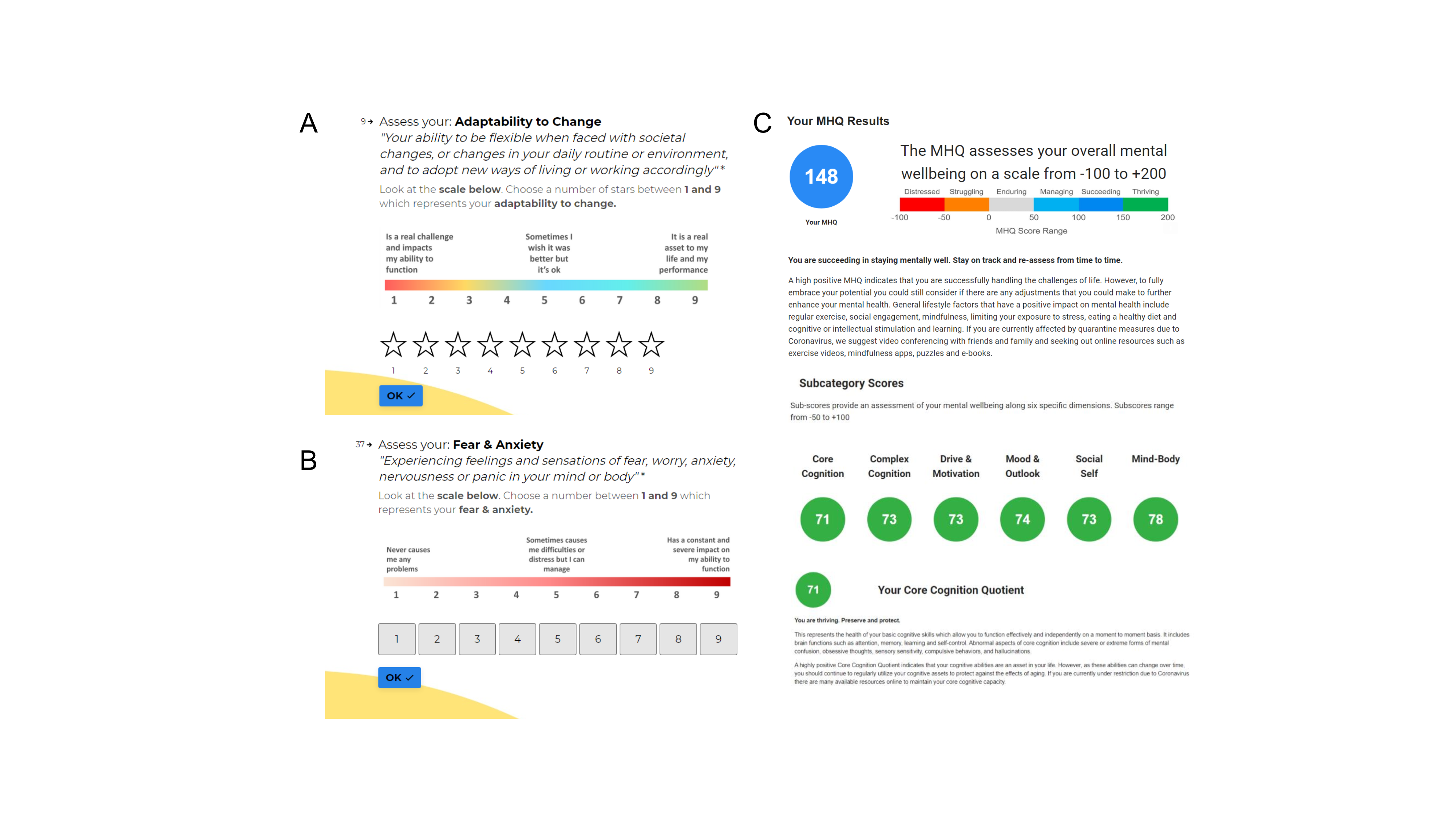

Supplement: Supplementary file 2 [file Image_1.TIF]
